# Percutaneous Suture - Closure Device Used for Iatrogenic Carotid Artery Injury Management: Case Report and Review of Literature

**DOI:** 10.1177/15385744231151557

**Published:** 2023-01-08

**Authors:** Yizhou Hu, Belal Al Khiami

**Affiliations:** 1 Division of Cardiovascular Medicine, 8784 University of California San Diego, La Jolla, CA, USA

**Keywords:** perclose pro glide device, vascular closure device, common carotid artery, hemostasis, iatrogenic injury

## Abstract

Accidental carotid artery injury is an uncommon but serious central venous catheter insertion complication. Hemostasis might not be readily achieved by manual compression; therefore, surgery or endovascular treatment remains the mainstay for accidental carotid artery injury. However, not all patients are suitable candidates for surgery.

Vascular closure devices are widely used in femoral arteries to achieve hemostasis and early ambulation. The use of vascular closure devices is occasionally reported in other vascular beds. Here we present a case of an iatrogenic left common carotid artery injury treated by vascular closure device, which is of help in the future management of this complication.

## Case Presentation

A 62-year-old Caucasian female with a past medical history of COVID-19 infection complicated by heart failure requiring an orthotopic heart transplant and known right internal jugular venous occlusion presented for elective routine heart biopsy via left internal jugular vein access. The case was complicated by accidental left common carotid access by introducing a 7F sheath which was determined later by pressure transduction from the pulmonary artery catheter. Venous access was initially performed by micro-puncture kit and thought to be confirmed by fluoroscopy with J-wire passing mid-line and curving as if traversing the right atrium and going towards with right ventricle.

The biopsy was canceled due to the complication. At this point, after a careful review of the patient’s case and discussion with treatment teams including heart transplant service, interventional cardiology and vascular surgery, decision was made to proceed with vascular closure devices attempt to achieve hemostasis immediately following the index procedure (Image A). Due to lack of the use of perioperative anticoagulation, manual hemostasis was considered but was not pursued due to low neck level access, known left internal carotid 50-69% stenosis with theoretically higher risk of stroke with prolonged compression and preference for more controlled intervention. After assuring the access site is in common carotid artery using ultrasound guidance which was already known to be free of disease based on recent carotid duplex studies, The Perclose ProGlide suture mediated closure device (Abbot vascular, Redwood city, CA) was deployed successfully in the left common carotid artery over a .035′ regular J tip wire and successful hemostasis was obtained. Light manual pressure was applied for 5 minutes following deployment. Deployment with gentle traction on the vessel wall was performed to reduce the risk of device failure or extending the vascular injury. After the procedure, an ultrasound was performed, which showed no evidence of pseudoaneurysm or arteriovenous fistula. Except for mild pain in the left neck, the patient had no focal neurological defect. The patient was admitted to intensive care unit (ICU) for close neurological observation over 24 hours. Aspirin 81 mg was continued.

**Image A.**
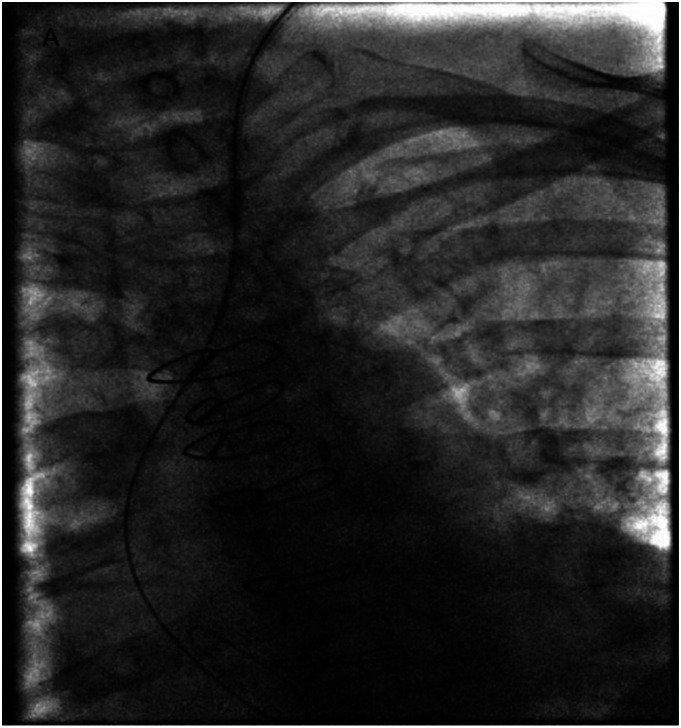
Catheter accidentally inserted into common carotid artery and aorta, mimicking the course of internal jugular vein and superior vena cava

Five days later, right heart catheterization with biopsy was successfully conducted with femoral access. The patient was safely discharged without complication. No major complications were detected during her stay in the hospital.

## Discussion

Carotid arterial puncture is one of the main complications of jugular venous catheter placement.^1^ Accidental iatrogenic arterial catheterization should be suspected on pulsatile or excessive backflow of blood.^2^ Following best practice access techniques like ultrasound guided access, micropuncture access needle utilization, pressure transducing and fluoroscopy use is crucial to reduce vascular access related complications.

In our case, unfortunately, multiple anatomical challenges increased this risk. This includes obesity with short neck anatomy, left rather than right internal jugular venous access and the quite tortuous ascending aorta mimicking the course of the left brachiocephalic and superior vena cava (Image A). In retrospect, transducing micropuncture access sheath pressure could have prevented upsizing the access sheath. This highlights the importance of following all best practices steps especially when there is any doubt.

Our case report, to our knowledge, is the third in literature to report the outcome of using suture based vascular closure device (VCD) to repair iatrogenic carotid injury. Excellent outcomes were reported in the two previous case reports.^3,4^

Open surgical approach is considered the gold standard for vascular injury repair and achieving hemostasis if manual compression is not feasible and safe.^5^ On the other hand, emerging endovascular approaches such VCD use, covered stent graft placement and balloon tamponade strategy provide a less invasive solutions^2,6,7^ but with less known safety and efficacy profile compared to surgical repair. High surgical risk patients particularly might benefit from these less invasive options.

VCD are predominantly used in common femoral arteries. Although off-label VCD use in the closure of alternative vascular access sites, such as subclavian, axillary, brachial, popliteal arteries,^2,6,8^ are occasionally reported with excellent outcomes. The widespread use of VCD in femoral arteries has been proved to significantly decrease the time to hemostasis, improve patient comfort, early mobilization and early discharge.^9-11^ Compared to manual compression, VCD demonstrates a similar complication rate, safety, and efficacy.^9^ There are two main types of VCD by mechanism, mechanical plug devices and arteriotomy edge to edge approximation devices. Mechanical plug devices are many and could use varied materials like collagen in the AngioSeal device (St. Jude Medical, St. Paul, MN) which also promotes thrombogenesis at access site to facilitate hemostasis while edge to edge approximation devices could use suture material like Perclose Proglide device (Abbot Vascular, Redwood City, CA) or Nitinol clip like StarClose device (Abbott vascular, Redwood City, CA).

In our case, surgery and endovascular treatment options were the mainstay of the treatment as hemostasis by manual compression only was not felt to be feasible and safe for the reasons mentioned previously in the case presentation section of this report. The decision to proceed with suture based VCD as a first strategy rather than operative was made as the patient was felt to be above average risk for operative repair and the lack of disease at access site by ultrasound vascular imaging. Also, the access sheath size was less than 9F. The lack of significant vascular disease is an important consideration to safely use such closure devices, so it is crucial to review any available baseline vascular studies and perform a baseline access angiogram or ultrasound study.

Suture based closure device was preferred over collagen plug device in this case as access could be maintained if device fails by keeping wire position, in which case, operative repair or other endovascular treatment choices can sought safely. Also, it has theoretically less embolization risk due to lack of loose material that could be entrapped inside the lumen and embolize.

VCD is known to be associated with complications such acute vessel closure, thrombosis, dissection, device failure and bleeding and operator should be able to recognize these complications and able to manage them. It is also crucial to ensure surgical back-up plans are in place for any complications should they occur as many of those can be only managed surgically.

To conclude, Our case provides added evidence of the safety, and efficacy of suture-based closure device use in managing iatrogenic carotid artery injury. However, further studies need to be done to avoid any potential reporting bias.

## APPENDIX

### List of abbreviations

intensive care unit (ICU)

vascular closure device (VCD)
